# Prognostic nutritional index (PNI) correlates with survival in head and neck cancer patients more precisely than other nutritional markers – real world data

**DOI:** 10.1007/s00405-024-08865-w

**Published:** 2024-08-06

**Authors:** Imre Uri, Angéla Horváth, László Tamás, Gábor Polony, Kornél Dános

**Affiliations:** 1 Department of Oto-Rhino-Laryngology and Head-Neck Surgery, Semmelweis University, Budapest, Hungary; 2 Department of Voice, Speech and Swallowing Therapy, Faculty of Health Sciences, Semmelweis University, Budapest, Hungary

**Keywords:** Nutrition, Screening, Body-mass index, Prognostic nutritional index, HPV, p16

## Abstract

**Purpose:**

The survival benefit with higher body mass index (BMI) of patients suffering from head and neck squamous cell carcinoma (HNSCC) is documented as *BMI paradox*. As the early re-nourishment of high-risk patients determine survival, we searched for a *nutritional status marker suitable for everyday screening*. Grouping patients based on the 8th Edition of TNM Classification, we investigated for the first time the candidate nutritional status markers among TNM8 subgroups, including the newly introduced p16 positive oropharyngeal squamous cell cancer (OPSCC) patients.

**Methods:**

We conducted a retrospective cohort study enrolling 661 patients and collecting anthropometric indices, laboratory parameters, clinical scores, nutritional risk scores. To discover the best one for screening survival analyses and correlation tests were executed.

**Results:**

By performing univariate Cox regression, we found three nutritional markers significantly correlating with overall survival (OS) and cancer specific survival (CSS): BMI at diagnosis, percent of weight loss over six months and prognostic nutritional index (PNI). The latter proved to be independent of tumor stage. p16 negative OPSCC patient’s OS and CSS did not correlate with BMI, but it did correlate with PNI and percent of weight loss. BMI was the only marker correlating with OS, only in stage 4 hypopharyngeal cancer patients. All three markers significantly correlated with survival among p16 positive oropharyngeal and glottic cancer patients.

**Conclusion:**

We found BMI, percent of weight loss and PNI good candidate markers for malnutrition. PNI proved to be superior in every aspect, enabling the treating physicians to discover high-risk patients in need of aggressive re-nourishment. The survival of supraglottic laryngeal squamous cancer patients seemed to be independent of these nutritional status markers, which observation should be a subject of further investigations.

## Introduction

The landscape of head-neck squamous cell carcinomas (HNSCC) has changed. Appropriate nourishment of high-risk patients is especially crucial regarding their overall survival (OS), cancer specific survival (CSS) and fitness to treatment. Optimal evaluation of nutritional status requires a marker which is sensitive and affordable enough for regular use. As laboratory tests became an integral part of patient workup, more prognostic and predictive factors are at reach.

Obesity is known as a cardiovascular and metabolic risk factor compromising life expectancy, but it unexpectedly brings survival benefit in HNSCC patients - known as body mass index (BMI) paradox [1–3].

Assessing BMI is one of the several methods of estimating nutritional status, which is defined as “the result between the nutritional intake received and the nutritional demands, and should allow for the utilization of nutrients to maintain reserves and compensate for losses.” [4] There are numerous markers: anthropometric indices, laboratory parameters, clinical scores, nutritional risk scores, questionnaires, body composition and dietary intake assessment [5]. It needs to be clarified which one is applicable for everyday screening of malnutrition. It should be easily accessible, should correlate with other nutritional status markers, have impact on OS and CSS, and it should be applicable for most tumor localizations and stages [6, 7].

Patients suffering from head-neck cancer form quite a diverse population. TNM Classification of Malignant Tumors 8th edition groups patients according to the site of origin, and in case of oropharyngeal cancers it distinguishes p16-positive and negative cancers, irrespective of HPV DNA status [8–11]. Nutritional status needs to be confirmed in this relatively new tumor group with distinct pathogenesis, patient age, social distribution, and better response to therapies [12–16].

Our aim was to find a sole nutritional status marker with powerful prognostic value to efficiently screen for high-risk patients. We intended to compare the predictive potency of the candidate markers on our whole HNSCC sample and in different TNM8 localizations (including p16 positive HNSCC patients as a new subgroup).

## Patients and methods

We performed a retrospective, cohort study. All patients enrolled were diagnosed with squamous cell carcinoma of the head and neck (HNSCC) between 2014 and 2023 at the Department of Oto-Rhino-Laryngology, Head and Neck Surgery, Semmelweis University, Budapest, Hungary, a tertial referral center treating patients from all over the country, therefore harboring the potential to represent the whole Hungarian HNSCC population. We included oral cavity, p16 positive and negative oropharyngeal, hypopharyngeal, supraglottic, glottic and subglottic laryngeal HNSCC patients and determined tumor stage following the UICC 8th TNM classification system.

Surgical samples were histologically processed by the Department of Pathology, Forensic and Insurance Medicine, Semmelweis University. To determine p16 status, immunohistochemistry with p16^INK4^-labeling was used, where test positivity was defined as at least 70% positive tumor cells.

We defined the term “diagnosis” as the date of biopsy taken from the tumor tissue.

Candidate nutritional status markers were assembled from systematic reviews related to this topic [17–20], as it follows:

- BMI at diagnostic sampling.
- BMI six months before diagnosis.
- percent of weight loss over six months before diagnosis.
- total lymphocyte count.
- hemoglobin.
- serum total protein.
- serum albumin.
- serum cholesterol.
- serum carbamide.
- serum creatinine.
- carbamide/creatinine ratio, calculated from the previous two markers.
- Prognostic Nutritional Index (PNI), calculated from total lymphocyte count and serum albumin.
- Nutritional Risk Index (NRI), calculated from serum albumin and percent of weight loss.
- Geriatric Nutritional Risk Index (GNRI), derived from serum albumin, actual and ideal weight (according to Lorentz-formula).
- Controlling Nutritional Status Score (CONUT), derived from total lymphocyte, serum albumin and cholesterol scores.

Anthropometric parameters (height, weight) were measured and weight loss (during the last half year) was asked and assessed before first tumor board presentation, which happened within a month after diagnostic sampling. All serum chemistry and hematology blood tests were collected in a range between one year before to eight weeks after diagnostic biopsy. Laboratory workup was done at the Department of Laboratory Medicine, Semmelweis University.

Carbamide and creatinine values are obtained from muscle and protein metabolism. These are used in the daily routine to assess renal function. To avoid the confounding effect of kidney failure, we excluded patients’ samples with carbamide over 7.2 mmol/liter and creatinine over 114.9 μmol/liter for men or 97.2 μmol/liter for women.

Carbamide/creatinine ratio is useful for assessing catabolism in normal renal function patients according to some sources [21]. We counted carbamide/creatinine ratio as: carbamide [mmol/liter] x 1000 / creatinine [μmol/liter].

After collecting height and weight at diagnosis and before it with at least six months, we calculated BMI at diagnosis, BMI before disease and percent of weight loss over six months. For BMI categories, the WHO classification was used: below 18.5: underweight; 18.5–24.9: normal weight; 25.0–29.9: overweight; over 29.9: obese. For weight loss, we divided it to mild (< 5%), moderate (5–10%) and severe extent (> 10%). (Table 1)

**Table 1:** Nutritional scores

	Body Mass Index	Percent of weight loss	Prognostic Nutritional Index	Nutritional Risk Index	Geriatric Nutritional Risk Index		Controlling Nutritional Status Score	
**abbreviation**	BMI	weight loss %	PNI	NRI	GNRI	**Ideal weight [kg]** **according to** **Lorentz-formula** **for men**: height [cm] − 100 - (height [cm] − 150) /4 **for women**: height [cm] − 100 - (height [cm] − 150) /2	CONUT	**albumin score**: 0 if alb. ≥ 35 gram/liter, 2 if alb.: 30–34.9 gram/liter, 4 if alb.: 25.0–29.9 gram/liter, 6 if alb.: < 25.0 gram/liter​ **cholesterol score**: 0 if chol. ≥ 4.66 mmol/liter, 1 if chol.: 3.62–4.65 mmol/liter, 2 if chol.: 2.59–3.61 mmol/liter, 3 if chol. < 2.59 mmol/liter **lymphocyte score**: 0 if lymph. ≥ 1.6 Giga/liter, 1 if lymph.: 1.20–1.59 Giga/liter, 2 if lymph.: 0.80–1.19 Giga/liter, 3 if lymph. < 0.8 Giga/liter
**count**	body weight [kg] / body height ^2 [m^2]	body weight now [kg] / body weight 6 months ago [kg] x 100	5 × lymphocyte count [Giga/liter] +​ serum albumin [gram/liter]	1.519 × serum albumin [gram/liter] + 41.7 × (actual / previous weight) [kg/kg]	1.489 × albumin [gram/liter] + 41.7 × (actual / ideal weight) [kg/kg]​		albumin score + cholesterol score + lymphocyte score	
**category**	**WHO categories**	**severity of weight loss**	**risk to malnutrition**:				**risk to malnutrition**:	
	obese if BMI ≥ 30	no weight loss	no if PNI ≥ 50	no if NRI ≥ 100	no if GNRI ≥ 98		no if CONUT: 0–1	
	overweight if BMI: 25-29.9	mild if weight loss < 5%	mild if PNI: 45-49.9	mild if NRI: 97-99.9	mild if GNRI: 92-97.9		mild if CONUT: 2–4	
	normal weight if BMI: 18.5–24.9	moderate if weight loss: 5–9.9%	moderate if PNI: 40-44.9	moderate if NRI: 83.5–96.9	moderate if GNRI: 82-91.9		moderate if CONUT: 5–8	
	underweight if BMI < 18.5	severe if weight loss ≥ 10%	severe if PNI < 40	severe if NRI < 83.5	severe if GNRI < 82		severe if CONUT: 9–12	

We calculated the following risk scores: Prognostic Nutritional Index (PNI), Nutritional Risk Index (NRI), Controlling Nutritional Status Score (CONUT)​ and Geriatric Nutritional Risk Index (GNRI). The latter is advantageous, if the patient was sarcopenic before the disease or did not remember to the extent of weight loss [22]. Their calculation and interpretation are presented in Table 1.

We assessed the Hungarian National Cancer Registry on 28th February 2023 as a censoring database and calculated OS in weeks by subtracting diagnosis date from it. Besides overall survival (OS), we also calculated cancer specific survival (CSS) to minimize the confounding effect of advanced age and concomitant diseases. For CSS, the tumor free status and presence of intercurrent disease were verified or rejected by reviewing the documentations of regular follow-up visits and imaging procedures in the internal computer database of the Semmelweis University.

Five-year survival was determined only if the patient’s diagnostic biopsy was at least five years before 28th February 2023.

For statistical analysis IBM SPSS Statistics 28.0 and TIBCO Statistica 14.0 were used.

Many of our variables did not show normal distribution (Kolmogorov-Smirnov and Shapiro-Wilk tests showed significant alteration from normal distribution), therefore analyses were performed by non-parametric tests: Chi-square tests, Spearman rank order correlations, univariate Cox-regressions, uni- and bivariate Cox proportional hazards regressions. The significance level is set to 5% in most tests, and to 1% in Spearman rank order correlations.

Written informed consents were obtained from all individual participants included in the study for data collection, processing, and storage before diagnostic sampling.

All procedures were in accordance with the ethical standards of the institutional and national research committee and with the 1964 Helsinki declaration and its later amendments. This research was approved by the Semmelweis University’s Regional, Institutional Scientific and Research Ethics Committee (SE TUKEB 105/2014).

## Results

Altogether 661 patients met the inclusion criteria. Investigating the descriptive statistics of the new TNM8 group, p16 positive oropharyngeal cancer patients were younger than p16 negative OPSCC or other HNSCC groups with mean ages 58, 63 and 63 years at diagnosis, respectively. Of p16 positive OPSCC patients, 42% had a regular smoking history and 20% did abuse alcohol at diagnosis, however, these numbers for p16 negative OPSCC and other HNSCC patients were 73%, 41%, and 66%, 44%, respectively (*p* < 0.001 for both tobacco and alcohol abuse, Chi-squares are 72.561 and 20.567).

Comparing stages of the HNSCC patients, most belonged to stage 4 (48%) while stage 3 group included 18%, stage 2 17% and stage 1 comprised 15% of patients, whereas 3% had incomplete staging. Further descriptive statistics are displayed in Tables 2, 3 and 4.

**Table 2:** Descriptive statistics: tumor characteristics. Data presentation: “number (% of known)”

Total		all groups	oral cavity	p14 neg. oropharynx	p16 pos. oropharynx	hypopharynx	supraglottic larynx	glottic larynx	subglottic larynx
		*661*	60	150	91	133	53	167	7
5-year survival	yes	*121 (39%)*	*11 (61%)*	*12 (17%)*	*12 (40%)*	*16 (23%)*	*14 (45%)*	*55 (61%)*	*1 (33%)*
	no	*193 (61%)*	*7 (39%)*	*60 (83%)*	*18 (60%)*	*54 (77%)*	*17 (55%)*	*35 (39%)*	*2 (67%)*
	shorter surveillance	*347*	*42*	*78*	*61*	*63*	*22*	*77*	*4*
Tumor	1	*102 (16%)*	10 (18%)	17 (12%)	11 (13%)	10 (8%)	6 (12%)	48 (29%)	0 (0%)
	2	*172 (27%)*	13 (23%)	41 (29%)	36 (41%)	25 (20%)	15 (29%)	41 (25%)	1 (14%)
	3	*129 (20%)*	14 (25%)	20 (14%)	17 (20%)	30 (23%)	15 (29%)	31 (19%)	2 (29%)
	4	*234 (37%)*	20 (35%)	63 (45%)	23 (26%)	63 (49%)	15 (29%)	46 (28%)	4 (57%)
	unknown	*24*	3	9	4	5	2	1	0
Node	0	*278 (44%)*	28 (47%)	41 (29%)	9 (10%)	33 (26%)	24 (47%)	137 (83%)	6 (86%)
	nodal met.	*361 (56%)*	31 (53%)	100 (71%)	77 (90%)	96 (74%)	27 (53%)	29 (17%)	1 (14%)
	unknown	*22*	1	9	5	4	2	1	0
Metastasis	0	*602 (94%)*	58 (98%)	125 (89%)	85 (97%)	121 (95%)	45 (88%)	161 (96%)	7 (100%)
	1	*39 (6%)*	1 (2%)	16 (11%)	3 (3%)	7 (5%)	6 (12%)	6 (4%)	0 (0%)
	unknown	*20*	1	9	3	5	2	0	0
Stage	1	*97 (15%)*	9 (16%)	6 (4%)	25 (29%)	4 (3%)	5 (10%)	48 (29%)	0 (0%)
	2	*110 (17%)*	10 (18%)	16 (11%)	32 (37%)	6 (5%)	8 (16%)	37 (22%)	1 (14%)
	3	*117 (18%)*	9 (16%)	17 (12%)	26 (30%)	22 (17%)	10 (%)	31 (19%)	2 (29%)
	4	*314 (49%)*	29 (51%)	102 (72%)	4 (5%)	97 (75%)	28 (20%)	50 (30%)	4 (57%)
	unknown	*23*	3	9	4	4	2	1	0

**Table 3:** Descriptive statistics: patient demographics. Data presentation: “number (% of known)”

Total		all groups	oral cavity	p14 neg. oropharinx	p16 pos. oropharinx	hypopharynx	supraglottic larynx	glottic larynx	subglottic larynx
		*661*	60	150	91	133	53	167	7
Sex	female	*133 (20%)*	18 (30%)	37 (25%)	27 (30%)	19 (14%)	13 (25%)	19 (11%)	0 (0%)
	male	*528 (80%)*	42 (70%)	113 (75%)	64 (70%)	114 (86%)	40 (75%)	148 (89%)	7 (100%)
	unknown	*0*	0	0	0	0	0	0	0
Age	< 50	*61 (9%)*	4 (7%)	9 (6%)	21 (23%)	13 (10%)	2 (4%)	12 (7%)	0 (0%)
	50–70	*477 (72%)*	33 (55%)	126 (84%)	60 (66%)	104 (78%)	42 (79%)	107 (64%)	5 (71%)
	> 70	*122 (18%)*	23 (38%)	15 (10%)	10 (11%)	16 (12%)	9 (17%)	47 (28%)	2 (29%)
	unknown	*1*	0	0	0	0	0	1	0
ECOG	0	*334 (58%)*	38 (67%)	66 (51%)	68 (77%)	55 (49%)	20 (43%)	84 (59%)	3 (50%)
	1	*170 (29%)*	11 (19%)	40 (31%)	17 (19%)	40 (36%)	15 (33%)	45 (32%)	2 (33%)
	2	*48 (8%)*	6 (11%)	13 (10%)	3 (3%)	11 (10%)	6 (13%)	8 (6%)	1 (17%)
	3	*26 (4%)*	2 (4%)	10 (8%)	0 (0%)	5 (4%)	5 (11%)	4 (3%)	0 (0%)
	4	*2 (0%)*	0 (0%)	0 (0%)	0 (0%)	1 (1%)	0 (0%)	1 (1%)	0 (0%)
	unknown	*81*	3	21	3	21	7	25	1
Tobacco use	never	*58 (9%)*	6 (11%)	7 (5%)	27 (36%)	5 (4%)	0 (0%)	13 (8%)	0 (0%)
	previous	*128 (21%)*	17 (32%)	23 (17%)	11 (14%)	26 (20%)	9 (17%)	41 (27%)	1 (17%)
	active	*425 (70%)*	30 (57%)	109 (78%)	38 (50%)	100 (76%)	44 (83%)	99 (65%)	5 (83%)
	unknown	*50*	7	11	15	2	0	14	1
Alcohol abuse	never	*243 (43%)*	29 (56%)	43 (36%)	50 (69%)	37 (30%)	18 (36%)	65 (45%)	1 (17%)
	previous	*60 (11%)*	6 (12%)	17 (14%)	4 (6%)	17 (14%)	6 (12%)	9 (6%)	1 (17%)
	active	*263 (46%)*	17 (33%)	61 (50%)	18 (25%)	68 (56%)	26 (52%)	69 (48%)	4 (67%)
	unknown	*95*	8	29	19	11	3	24	1
Diabetes	not known	*570 (88%)*	51 (85%)	131 (90%)	78 (90%)	118 (90%)	47 (89%)	139 (85%)	6 (100%)
	known	*76 (12%)*	9 (15%)	14 (10%)	9 (10%)	13 (10%)	6 (11%)	25 (15%)	0 (0%)
	unknown	*15*	0	5	4	2	0	3	1

**Table 4:** Descriptive statistics: nutritional status markers. Data presentation: “number (% of known)”

		all groups	oral cavity	p14 neg. oropharinx	p16 pos. oropharinx	hypopharynx	supraglottic larynx	glottic larynx	subglottic larynx
BMI	obese	*98 (16%)*	11 (18%)	15 (10%)	23 (26%)	14 (11%)	7 (14%)	27 (17%)	1 (14%)
	overweight	*153 (24%)*	15 (25%)	26 (18%)	28 (32%)	21 (17%)	11 (22%)	51 (32%)	1 (14%)
	normal	*309 (49%)*	25 (42%)	84 (58%)	32 (37%)	72 (58%)	21 (43%)	72 (46%)	3 (43%)
	underweight	*71 (11%)*	9 (15%)	20 (14%)	4 (5%)	18 (14%)	10 (20%)	8 (5%)	2 (29%)
	unknown	*30*	0	5	4	8	4	9	0
weight loss %	no	*305 (57%)*	*35 (61%)*	*52 (40%)*	*50 (68%)*	*51 (48%)*	*16 (39%)*	*98 (77%)*	*3 (75%)*
	< 5%	*55 (10%)*	*3 (5%)*	*18 (14%)*	*8 (11%)*	*10 (9%)*	*4 (10%)*	*12 (9%)*	*0 (0%)*
	5–10%	*86 (16%)*	*9 (16%)*	*29 (22%)*	*10 (14%)*	*20 (19%)*	*11 (27%)*	*6 (5%)*	*1 (25%)*
	> 10%	*93 (17%)*	*10 (18%)*	*30 (23%)*	*5 (7%)*	*26 (24%)*	*10 (24%)*	*12 (9%)*	*0 (0%)*
	unknown	*175*	6	37	27	32	16	53	4
PNI (risk to malnutrition)	no	*67 (40%)*	3 (33%)	7 (20%)	9 (60%)	23 (52%)	3 (15%)	21 (50%)	1 (100%)
	mild	*26 (16%)*	1 (11%)	9 (26%)	2 (13%)	6 (14%)	5 (25%)	3 (7%)	0 (0%)
	moderate	*36 (22%)*	3 (33%)	8 (23%)	3 (20%)	10 (23%)	5 (25%)	7 (17%)	0 (0%)
	severe	*37 (22%)*	2 (22%)	11 (31%)	1 (7%)	5 (11%)	7 (35%)	11 (26%)	0 (0%)
	unknown	*495*	51	115	76	89	33	125	6
NRI (risk to malnutrition)	no	*48 (35%)*	3 (38%)	8 (26%)	7 (70%)	13 (35%)	2 (13%)	15 (43%)	0 (0%)
	mild	*8 (6%)*	0 (0%)	1 (3%)	0 (0%)	2 (5%)	2 (13%)	3 (9%)	0 (0%)
	moderate	*58 (43%)*	4 (50%)	14 (45%)	3 (30%)	20 (54%)	7 (47%)	10 (29%)	0 (0%)
	severe	*22 (16%)*	1 (13%)	8 (26%)	0 (0%)	2 (5%)	4 (27%)	7 (20%)	0 (0%)
	unknown	*525*	52	119	81	96	38	132	7
GNRI (risk to malnutrition)	no	*89 (57%)*	5 (56%)	20 (59%)	8 (67%)	24 (60%)	7 (39%)	24 (59%)	1 (100%)
	mild	*21 (14%)*	2 (22%)	1 (3%)	2 (17%)	7 (18%)	4 (22%)	5 (12%)	0 (0%)
	moderate	*23 (15%)*	1 (11%)	5 (15%)	2 (17%)	6 (15%)	2 (11%)	7 (17%)	0 (0%)
	severe	*22 (14%)*	1 (11%)	8 (24%)	0 (0%)	3 (8%)	5 (28%)	5 (12%)	0 (0%)
	unknown	*506*	51	116	79	93	35	126	6
CONUT (risk to malnutrition)	no	*12 (41%)*	0 (0%)	0 (0%)	4 (80%)	3 (60%)	3 (60%)	2 (33%)	0 (0%)
	mild	*9 (31%)*	0 (0%)	4 (50%)	1 (20%)	1 (20%)	1 (20%)	2 (33%)	0 (0%)
	moderate	*7 (24%)*	0 (0%)	3 (38%)	0 (0%)	1 (20%)	1 (20%)	2 (33%)	0 (0%)
	severe	*1 (3%)*	0 (0%)	1 (13%)	0 (0%)	0 (0%)	0 (0%)	0 (0%)	0 (0%)
	unknown	*492*	60	142	86	48	48	161	7

**Graph 1:**
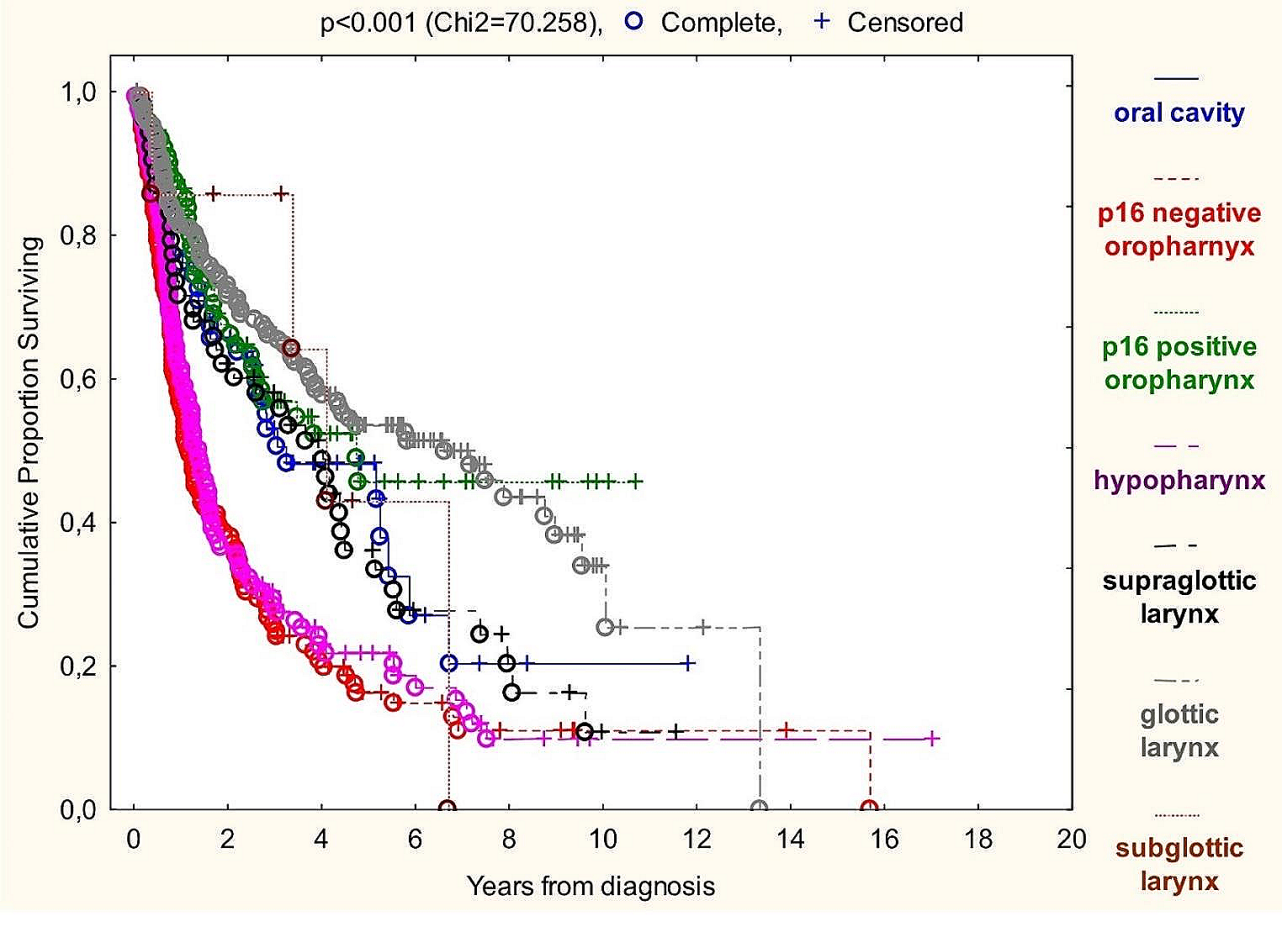
Kaplan-Meier overall survival analysis of TNM8 HNSCC groups

Analyzing Kaplan-Meier curves (Graph 1), the best OS among TNM8 groups was associated with glottic laryngeal and p16 positive oropharyngeal cancer patients, while p16 negative oropharyngeal and hypopharyngeal carcinomas had the most devastating prognosis. Subglottic patients’ OS was inconclusive due to the small sample size.

Median survival times of patients with at least five-year surveillance were the following: 7.0 months assuming the whole sample, 8.0 months for oral cavity, 4.4 months for p16 negative and 7.4 months for p16 positive oropharyngeal, 40.6 months for hypopharyngeal, 8.5 months for supraglottic-, 9.5 for glottic- and 11.8 for subglottic laryngeal squamous cell carcinoma patients.

Kolmogorov-Smirnov test proved deviation from normal distribution in the case of cholesterol, albumin, PNI, NRI, GNRI with *p* > 0.20.

Spearman rank order test have proven correlation at 1% significance level between BMI and weight loss, and between PNI, NRI, GNRI and CONUT.

We performed univariate Cox-regressions for overall survival and cancer specific survival in each TNM8 group (Tables 5 and 6). If the results of both analyses were significant with unidirectional risk ratios, we only indicated results of the more widely used OS, otherwise we presented both OS and CSS.

**Table 5:** Univariate Cox-regression with overall survival. (x: insufficient data amount), if *p* < 0.05, RR = risk ratio, CI = confidence interval at *p* = 0.05

	all groups		oral cavity		p16 negative oropharynx		p16 positive oropharynx		hypopharynx		supraglottic larynx	glottic larynx		subglottic larynx	
	*p*	RR (CI)	*p*	RR (CI)	*p*	RR (CI)	*p*	RR (CI)	*p*	RR (CI)	*p*	*p*	RR (CI)	*p*	
**BMI at diagnosis**	**< 0.001**	**0.938 (0.918–0.959)**	**0.500**		**0.970**		**0.013**	**0.917 (0.856–0.982)**	**0.010**	**0.941 (0.897–0.986)**	**0.530**	**0.001**	**0.914 (0.865–0.966)**	0.874	
BMI before disease	0.003	0.965 (0.942–0.988)	0.981		0.149		0.323		0.028	0.947 (0.902–0.994)	0.674	0.075		0.425	
**weight loss %**	**< 0.001**	**1.050 (0.036–1.064)**	**0.391**		**0.016**	**1.031 (1.006–1.056)**	**0.001**	**1.067 (1.027–1.108)**	**0.264**		**0.449**	**< 0.001**	**1.128 (1.084–1.174)**	**0.838**	
lymphocyte	< 0.001	0.740 (0.628–0.871)	0.028	0.554 (0.327–0.939)	0.176		0.108		0.216		0.651	0.042	0.663 (0.446–0.985)	0.878	
hemoglobin	< 0.001	0.983 (0.977–0.988)	0.204		0.295		0.005	0.973 (0.953–0.992)	< 0.001	0.981 (0.971–0.992)	0.239	< 0.001	0.969 (0.955–0.985)	0.366	
total protein	0.036	0.980 (0.961–0.999)	0.018	0.898 (0.821–0.981)	0.436		0.037	0.893 (0.803–0.993)	0.535		0.525	0.189		x	
albumin	0.001	0.956 (0.929–0.983)	0.028	0.852 (0.739–0.983)	0.041	0.940 (0.886–0.998)	0.230		0.959		0.886	0.001	0.912 (0.862–0.965)	x	
carbamide	< 0.001	0.818 (0.753–0.887)	0.484		0.488		0.199		0.076		0.227	0.002	0.746 (0.622–0.894)	0.947	
creatinine	< 0.001	0.983 (0.977–0.990)	0.757		0.974		0.101		0.061		0.762	< 0.001	0.966 (0.948–0.983)	0.224	
carbamid/creatinine ratio	0.455		0.293		0.457		0.216		0.743		0.083	0.858		0.494	
cholesterol	0.037	0.822 (0.683–0.988)	x		0.012	0.634 (0.444–0.905)	0.860		0.012	0.585 (0.386–0.888)	0.293	0.922		x	
**PNI**	**< 0.001**	**0.947 (0.926–0.970)**	**0.022**	**0.899 (0.820–0.985)**	**0.003**	**0.918 (0.867–0.972)**	**0.019**	**0.871 (0.776–0.978)**	**0.792**		**0.644**	**< 0.001**	**0.901 (0.859–0.946)**	**x**	
NRI	0.003	0.969 (0.949–0.989)	0.061		0.922		0.128		0.347		0.514	0.001	0.934 (0.898–0.971)	x	
GNRI	< 0.001	0.968 (0.954–0.982)	0.135		0.411		0.205		0.349		0.301	< 0.001	0.937 (0.908–0.965)	x	
CONUT	0.031	1.246 (1.020–1.522)	x		0.001	2.055 (1.341–3.150)	0.990		0.912		0.321	0.006	1.825 (1.188–2.801)	x	

**Table 6:** Univariate Cox-regression with cancer specific survival. (x: insufficient data amount), if *p* < 0.05, RR = risk ratio, CI = confidence interval at *p* = 0.05

	all groups		oral cavity		p16 negative oropharynx		p16 positive oropharynx		hypopharynx		supraglottic larynx	glottic larynx		subglottic larynx	
	*p*	RR (CI)	*p*	RR (CI)	*p*	RR (CI)	*p*	RR (CI)	*p*	RR (CI)	*p*	*p*	RR (CI)	*p*	
**BMI at diagnosis**	**< 0.001**	**0.922 (0.899–0.948)**	**0.869**		**0.747**		**0.008**	**0.900 (0.832–0.973)**	**0.003**	**0.917 (0.865–0.971)**	**0.257**	**< 0.001**	**0.860 (0.798–0.925)**	**0.504**	
BMI before disease	< 0.000	0.952 (0.926–0.980)	0.970		0.320		0.177		0.010	0.922 (0.867–0.980)	0.789	0.036	0.922 (0.855–0.995)	0.495	
**weight loss %**	**< 0.001**	**1.058 (1.043–1.076)**	**0.018**	**1.058 (1.001–1.108)**	**0.047**	**1.028 (1.000-1.057)**	**0.002**	**1.067 (1.024–1.111)**	**0.093**		**0.058**	**< 0.001**	**1.137 (1.086–1.190)**	**0.863**	
lymphocyte	< 0.001	0.709 (0.585–0.860)	0.058		0.325		0.213		0.398		0.993	0.005	0.473 (0.282–0.793)	0.364	
hemoglobin	< 0.001	0.980 (0.973–0.986)	0.890		0.040	0.986 (0.973–0.999)	0.046	0.978 (0.956- 1.000)	< 0.001	0.974 (0.963–0.986)	0.676	< 0.001	0.957 (0.940–0.974)	0.350	
total protein	0.114		0.343		0.618		0.005	0.857 (0.770–0.956)	0.599		0.322	0.297		x	
albumin	0.002	0.950 (0.920–0.981)	0.431		0.065		0.130		0.355		0.86ö	0.017	0.918 (0.856–0.985)	x	
carbamide	< 0.001	0.804 (0.720–0.885)	0.523		0.726		0.654		0.013	0.799 (0.668–0.954)	0.142	0.005	0.722 (0.577–0.904)	0.682	
creatinine	< 0.001	0.984 (0.977–0.992)	0.539		0.936		0.195		0.158		0.935	0.008	0.970 (0.948–0.992)	0.408	
carbamid/creatinine ratio	0.290		0.353		0.801		0.070		0.301		0.078	0.320		0.471	
cholesterol	0.107		x		0.012	0.608 (0.414–0.895)	0.683		0.065		0.152	0.388		x	
**PNI**	**< 0.001**	**0.942 (0.918–0.967)**	**0.277**		**0.006**	**0.917 (0.861–0.976)**	**0.021**	**0.861 (0.758–0.978)**	**0.485**		**0.948**	**< 0.001**	**0.893 (0.845–0.943)**	**x**	
NRI	< 0.001	0.960 (0.938–0.983)	0.751		0.662		0.060		0.856		0.307	0.002	0.931 (0.889–0.974)	x	
GNRI	< 0.001	0.960 (0.945–0.975)	0.967		0.365		0.562		0.076		0.063	< 0.001	0.925 (0.894–0.956)	x	
CONUT	0.140		x		0.002	2.176 (1.326–3.570)	0.129		0.865		0.444	0.775		x	

**Graph 2:**
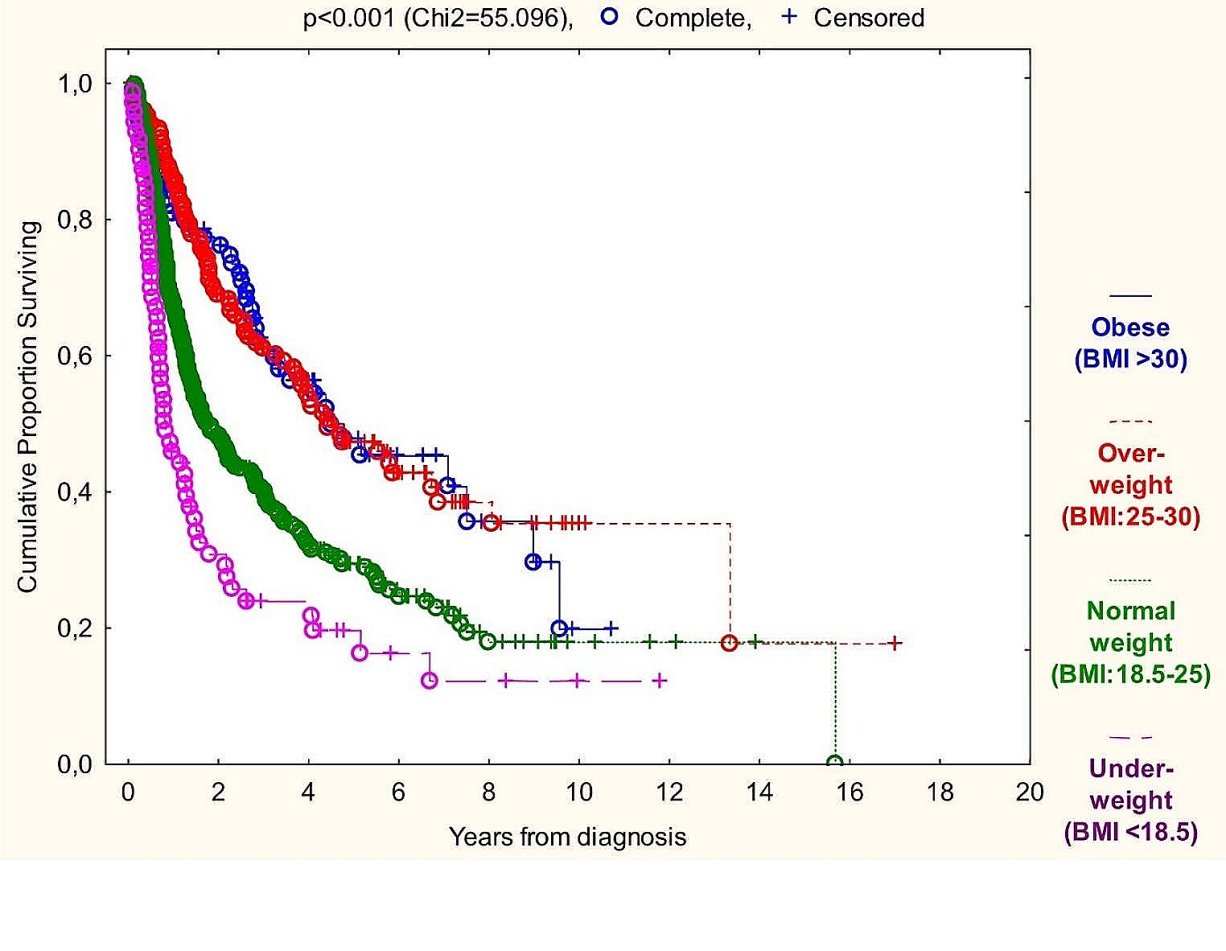
Kaplan-Meier overall survival analysis of BMI groups

Graph 2 with p_Chi2_ <0.001 underlines the BMI paradox in for the whole head and neck squamous cell cancer sample, but according to univariate Cox-regressions of TNM8 groups (Tables 5 and 6), oral cavity and p16 negative oropharyngeal cancer patients’ OS and CSS is independent of BMI status. Surprisingly p16 positive OPSCC fits in the row of “average” HNSCC behavior in this aspect.

Carbamide/creatinine ratio as theoretical catabolism marker did not affect OS or CSS. Hemoglobin strongly correlated with survival. Several parameters were significant only on the whole sample, but not in smaller subgroups: BMI before diagnosis (*p* = 0.003, RR = 0.965), total lymphocyte count (*p* < 0.001, RR = 0.740), serum albumin (*p* = 0.001, RR = 0.956), total protein (*p* = 0.036, RR = 0.980), cholesterol (*p* = 0.037, RR = 0.822), carbamide (*p* < 0.001, RR = 0.818), creatinine (*p* < 0.001, RR = 0.983), NRI (*p* = 0.003, RR = 0.969), GNRI (*p* < 0.001, RR = 0.968) and CONUT scores (*p* = 0.031, RR = 1.246) are of this assessment.

**Graph 3:**
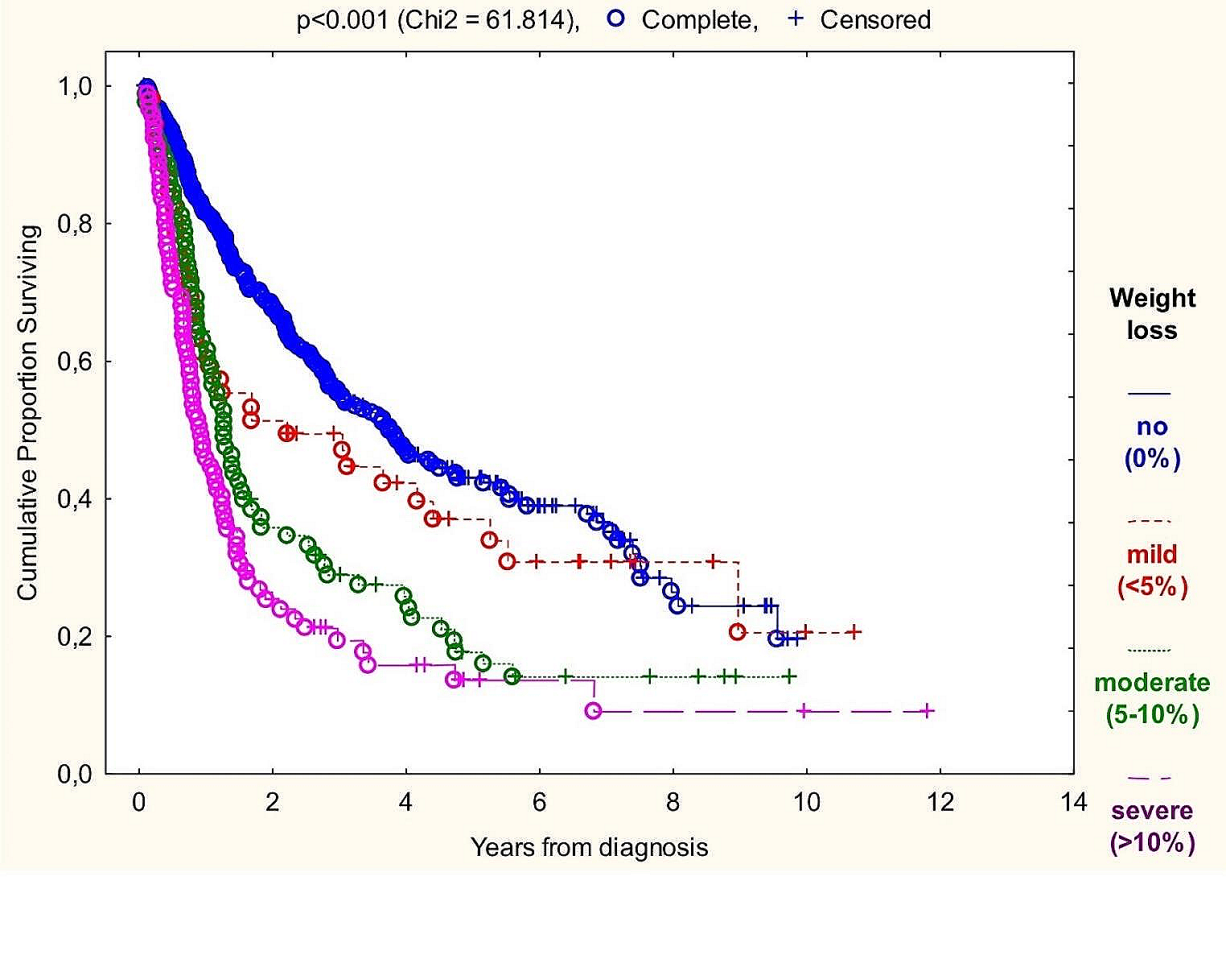
Kaplan-Meier overall survival analysis based on percent of weight loss

**Graph 4:**
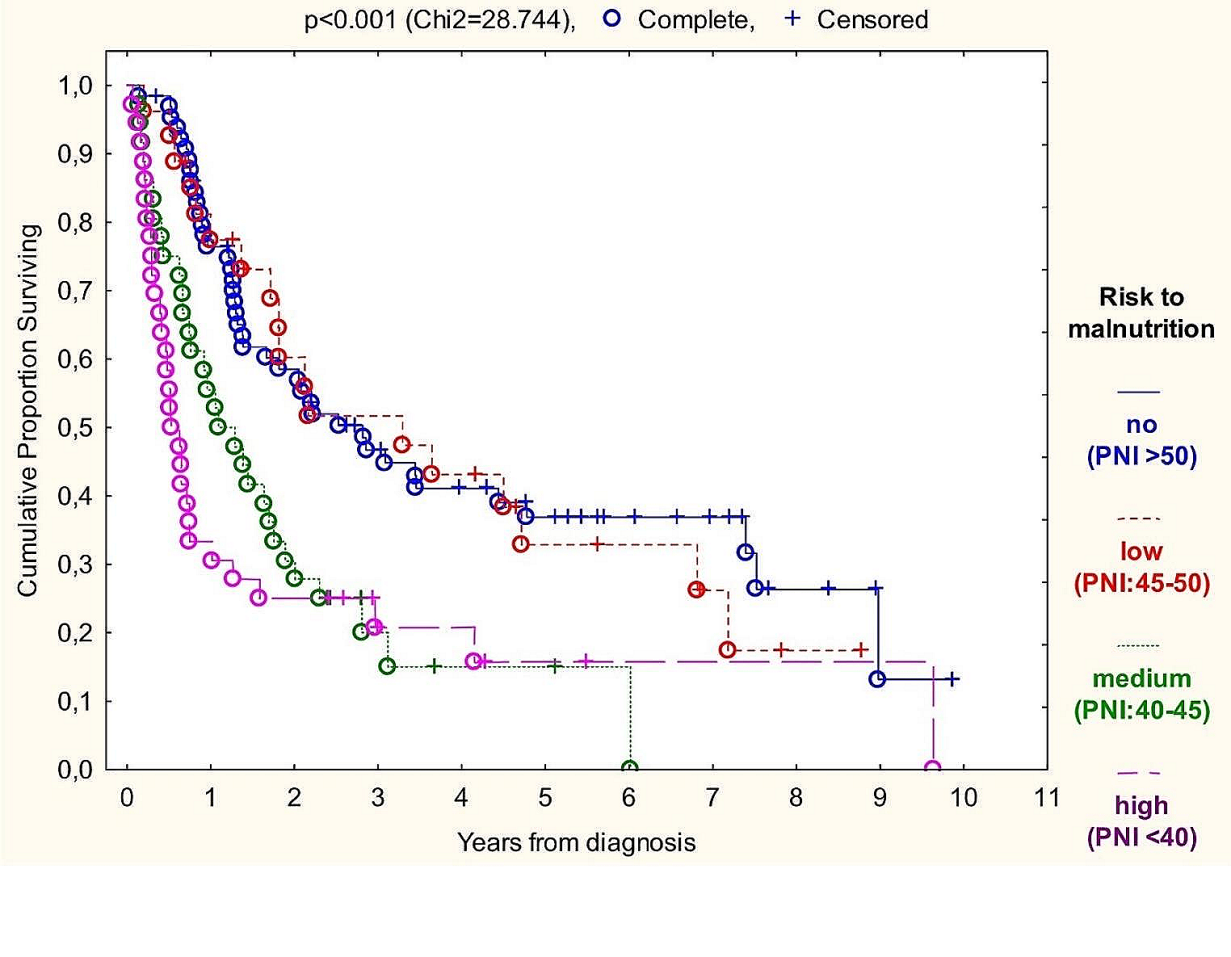
Kaplan-Meier overall survival analysis of PNI groups

The markers correlating with survival even in subgroups were BMI at diagnosis, percent of weight loss over six months and prognostic nutritional index (PNI) with risk ratio of 0.938 (*p* < 0.001), 1.050 (*p* < 0.001) and 0.947 (*p* < 0.001) on the total sample, respectively (Graphs 2, 3 and 4). Performing univariate Cox-regressions revealed the following:

- Only PNI correlated with oral cavity cancer patients’ OS (*p* = 0.022, RR = 0.899) and percent of weight loss correlated with their CSS alone (*p* = 0.018, RR = 1.058).
- Percent of weight loss (*p* = 0.016, RR = 1.031) and PNI (*p* = 0.003, RR = 0.918) correlated with p16 negative OPSCC patients’ OS.
- All three correlated with p16 positive OPSCC patients’ OS: BMI at diagnosis (*p* = 0.013, RR = 0.917), percent of weight loss (*p* = 0.001, RR = 1.067), PNI (*p* = 0.019, RR = 0.871).
- Only BMI at diagnosis correlated with hypopharyngeal cancer patients’ OS (*p* = 0.010, RR = 0.941). Only in stage 4 did BMI prove to significantly affect OS (*p* = 0.022, RR = 0.937).
- All three correlated with glottic laryngeal patients’ OS: BMI at diagnosis (*p* = 0.001, RR = 0.914), percent of weight loss (*p* < 0.001, RR = 1.128), PNI (*p* < 0.001, RR = 0.901).
- BMI at diagnosis and percent of weight loss did not correlate with subglottic patients’ OS. Sample size was not sufficient to run Cox regression with PNI.
- None of any investigated nutritional status markers correlated significantly with supraglottic laryngeal cancer patients’ survival.

Different groups owe different distribution of disease severity. To eliminate this confounder effect, we used uni- and bivariate Cox proportional hazards regression (Table 7). Stage 4 patients are set as baseline for risk evaluation, as this have the most considerable impact on all tests results, being the largest sample group (with 48% of patients). As BMI and percent of weight loss are strongly related (Spearman’s rank correlation coefficient is -0.452, *p* < 0.01) we could not perform bivariate regression with these markers. R^2^ values indicating goodness of fitting are distributed from 0.354 to 0.462. In the case of percent of weight loss and BMI categories, their prognostic value depended on tumor stage (as it has proven a risk factor in the regression model). PNI category’s predictive effect was independent of stage making it the most suitable nutritional status marker for everyday screening.

**Table 7:** Cox proportional hazards regression model based on cancer specific survival. Marker categories (no, low, medium, or high-risk to malnutrition) are the covariates, and stages according to TNM8 are the factors. Stage 4 is set as baseline

		univariate Cox proportional hazards regression			bivariate Cox proportional hazards regression	
		BMI category	percent of weight loss category	PNI category	BMI and PNI categories	percent of weight loss and PNI categories
R^2^		0.462	0.488	0.354	0.450	0.353
BMI category	*p*	< 0.001			0.003	
	Hazard ratio if *p* < 0.05 (confidence interval)	**1.398** **(1.199–1.630)**			**1.603** **(1.175–2.188)**	
percent of weight loss category	*p*		< 0.001			0.114
	Hazard ratio if *p* < 0.05 (confidence interval)		**1.243** **(1.115–1.387)**			
PNI category	*p*			< 0.001	< 0.001	0.003
	Hazard ratio if *p* < 0.05 (confidence interval)			**1.438** **(1.193–1.734)**	**1.429** **(1.181–1.731)**	**1.376** **(1.112–1.701)**
stage 1	*p*	**< 0.001**	**< 0.001**	0.053	0.249	0.167
	Hazard ratio if *p* < 0.05 (confidence interval)	**0.088** **(0.041–0.190)**	**0.085** **(0.035–0.204)**			
stage 2	*p*	**0.029**	0.229	0.718	0.954	0.690
	Hazard ratio if *p* < 0.05 (confidence interval)	**0.228** **(0.150–0.349)**				
stage 3	*p*	**0.041**	0.180	0.830	0.683	0.722
	Hazard ratio if *p* < 0.05 (confidence interval)	**0.448** **(0.321–0.625)**				

Note: bivariate Cox proportional hazards regression with BMI and percent of weight loss category is not amenable due to their correlated status (at 1% significance level)

Bivariate Cox proportional regression proved BMI and PNI both affect survival when applied together. (R^2^ = 0.450, p_BMI_=0.003, RR_BMI_=1.603, p_PNI_<0.001, RR_PNI_=1.429)

After running Receiver Operating Characteristic (ROC) analysis, we have found similar sensitivity and specificity properties of the observed parameters determining five-year survival. PNI proves the best performance (area under ROC curve = 0.704, *p* = 0.005), followed by BMI at diagnosis (area under ROC curve = 0.686, *p* = 0.010) and percent of weight loss (area under ROC curve = 0.324. As it negatively correlates with OS, we have to divide it from 1 to be comparable with other parameters, which equals to 0.676. *p* = 0.015).

## Discussion

There is a well-documented tendency towards malnutrition in the patient population suffering from head-neck squamous cancer [23–26] Disorders can be caused by the tumor and the treatment as well [27]. Dysphagia (sensation of having difficulty with swallowing) could develop by direct swallowing obstruction, innervational damage or xerostomia [28, 29]. Odynophagia (painful swallowing) and frequent aspiration could result in eating aversion and recurrent pneumoniae [27, 30]. Loss of appetite and explicit tumor metabolism lead to catabolic energy mobilization and cachexia [31]. The characteristic HNSCC patient is from poor socioeconomical group, some tend to spend on alcohol and tobacco rather than on a balanced, sufficient diet [32]. Poor oral hygiene (lack of bite and chewing function) also makes eating difficult. The initial nutritional status determines the patient’s suitability to the curative treatment.

Obesity is known as a cardiovascular and metabolic risk factor, but it unexpectedly brings a survival benefit in HNSCC patients - known as BMI paradox. Stepping forward, we searched for a nutritional status marker good enough for everyday screening to efficiently filter out high-risk patients and get the opportunity for early and intensive re-nourishment. This screening marker had to fulfill the following requirements:

- Affordable for routine use.
- Correlates with other nutritional status markers.
- Has a considerable effect on overall survival (OS) and cancer specific survival (CSS).
- Applicable for most patient subgroups.
- Independently good predictor in every tumor stage defined by TNM8.

We run a retrospective analysis on 661 patients suffering from HNSCC.

Nearly all observed markers have prognostic value to some extent in the whole sample, but only four have proven to impact OS and CSS in nearly all TNM8 groups: BMI at diagnostic sampling, percent of weight loss in six months, Prognostic Nutritional Index (PNI) and hemoglobin.

Hemoglobin is routinely screened, but as the etiology can be quite diverse beyond poor nutrition [17]. It is corrected by substitution of iron, vitamin-B12, folic acid or in severe case by blood transfusion, not by re-nourishment. Considering these reasons, we did not count it to the candidate nutritional status markers for screening.

We observed the Body Mass Index paradox (meaning that obese patients have better prognosis) in most TNM8 groups, except for in the case of oral cavity and p16 negative OPSCC, supra- and subglottic laryngeal cancer patients. We confirmed that BMI paradox does occur in the case of p16 positive OPSCC patients [15]. This follows the tendency observed in many previous studies [1, 2]. A research has found greater association between nutritional status and OS in HPV-induced cancers [33], but another has claimed it independent of HPV status [34]. A study have found obesity associated with higher risk of non-HPV HNSCC development [35]. PNI is a better choice to assess nutritional status of p16 negative OPSCC [36].

According to Cox proportional hazards regression, both percent of weight loss and BMI has different prognostic value in different TNM8 stages, which make them inconclusive and less fit for screening.

As for Prognostic Nutritional Index it fulfills every requirement for the ideal screening listed above. Other studies underly our findings related to the superiority of PNI among nutritional status markers [19, 36–41].

Comparing TNM8 groups, PNI correlated with OS and percent of weight loss correlated with CSS in oral cavity cancer patients. p16 negative oropharyngeal cancer patient’s survival did not correlate with BMI, but did with PNI and percent of weight loss. All three markers influence survival significantly among p16 positive oropharyngeal and glottic cancer patients, whereas supraglottic laryngeal cancer patients’s outcome showed no correlation with the markers mentioned. Among subglottic laryngeal originating cancer patients, neither BMI nor percent of weight loss influenced OS or CSS, and there was not enough element number to judge the effect of PNI. We found BMI the only marker affecting survival in the case of hypopharyngeal cancer patients, but only in stage 4.

Re-nourishing is essential is high-risk patients, which should start as soon as possible. The recommendation is 30–35 kcal/kg daily energy intake [42]. If amenable, oral route is preferred, but if due to dysphagia, odynophagia or malabsorption it is not sufficient for the daily intake, we should not fear of invasive enteral, or initial parenteral feeding. Prolonged wearing of nasogastric tube is discomforting, so prophylactic percutaneous or surgical gastrectomy is advised, as it improves survival outcomes [43]. Chemoradiation may worsen the symptoms of dysphagia and odynophagia, therefore long-term feeding is recommended [44].

## Conclusion

We found that Prognostic Nutritional Index (PNI) is the optimal nutritional status marker for identifying high-risk patients in most tumor localizations. It strongly correlates with overall survival, cancer specific survival and is unbiased of tumor stages defined by TNM8. This observation should be confirmed in prospective, cohort studies.

Body Mass Index and percent of weight loss are acceptable markers in case of PNI’s unavailability, but these have several limitations. In the case of oral cavity originating and p16 negative oropharyngeal squamous cell cancer patients, overall- and cancer specific survival is irrespective of BMI. BMI and percent of weight loss have different prognostic values in different tumor stages defined by TNM8. BMI and percent of weight loss strongly correlate, making it futile to measure both, whereas counting PNI when knowing BMI provides additive information about nutritional status.

The survival of supra- and subglottic laryngeal squamous cancer patients seem to be independent of nutritional status, which is not studied yet, needs to be confirmed.

## References

[1] Hobday S et al (2023) The body Mass Index Paradox in Head and Neck Cancer: a systematic review and Meta-analysis. Nutr Cancer 75(1):48–60. 10.1080/01635581.2022.2102659 35959747 10.1080/01635581.2022.2102659

[2] Hicks DF et al (2018) Impact of obesity on outcomes for patients with head and neck cancer. Oral Oncol 83:11–17. 10.1016/j.oraloncology.2018.05.027 30098765 10.1016/j.oraloncology.2018.05.027

[3] Gama RR et al (2017) Body mass index and prognosis in patients with head and neck cancer. Head Neck 39(6):1226–1233. 10.1002/hed.24760 28323362 10.1002/hed.24760

[4] Fernández-Lázaro D, Seco-Calvo J (2023) Nutrition, nutritional status and functionality. Nutrients 15(8):1944. 10.3390/nu15081944 10.3390/nu15081944 PMC10142726 37111162

[5] Prevost V et al (2014) Assessment of nutritional status and quality of life in patients treated for head and neck cancer. European annals of Otorhinolaryngology. Head Neck Dis 131(2):113–120. 10.1016/j.anorl.2013.06.007 10.1016/j.anorl.2013.06.007 24657191

[6] De Vries J et al (2013) Markers for nutrition studies: review of criteria for the evaluation of markers. Eur J Nutr 52(7):1685–1699. 10.1007/s00394-013-0553-3 23955424 10.1007/s00394-013-0553-3

[7] Calder PC et al (2017) Improving selection of markers in nutrition research: evaluation of the criteria proposed by the ILSI Europe marker validation initiative. Nutr Res Rev 30(1):73–81. 10.1017/s0954422416000263 28202104 10.1017/S0954422416000263

[8] Golusiński P et al (2017) Is immunohistochemical evaluation of p16 in oropharyngeal cancer enough to predict the HPV positivity? Rep Practical Oncol Radiotherapy 22(3):237–242. 10.1016/j.rpor.2017.01.003 10.1016/j.rpor.2017.01.003 PMC5403803 28461789

[9] Mehanna H et al (2023) Prognostic implications of p16 and HPV discordance in oropharyngeal cancer (HNCIG-EPIC-OPC): a multicentre, multinational, individual patient data analysis. Lancet Oncol 24(3):239–251. 10.1016/s1470-2045(23)00013-x 36796393 10.1016/S1470-2045(23)00013-X

[10] Brauswetter D et al (2017) p16(INK4) expression is of prognostic and predictive value in oropharyngeal cancers independent of human papillomavirus status: a Hungarian study. Eur Arch Otorhinolaryngol 274(4):1959–1965. 10.1007/s00405-016-4412-8 27999998 10.1007/s00405-016-4412-8

[11] Huang SH, O’Sullivan B (2017) Overview of the 8th edition TNM classification for head and neck cancer. Curr Treat Options Oncol 18(7):40. 10.1007/s11864-017-0484-y 28555375 10.1007/s11864-017-0484-y

[12] King B, Subramaniam R, Sanli Y (2018) Heterogeneity of HPV positive and negative oropharyngeal squamous cell carcinoma by FDG PET/CT. J Nucl Med 59(supplement 1):632–63229419475

[13] Fakhry C et al (2008) Improved survival of patients with human papillomavirus–positive head and neck squamous cell carcinoma in a prospective clinical trial. J Natl Cancer Inst JNCI(4):261–269. 10.1093/jnci/djn011 10.1093/jnci/djn011 18270337

[14] Lassen P et al (2009) Effect of HPV-associated p16INK4A expression on response to radiotherapy and survival in squamous cell carcinoma of the head and neck. J Clin Oncol 27(12):1992–1998. 10.1200/JCO.2008.20.2853 19289615 10.1200/JCO.2008.20.2853

[15] Albergotti WG et al (2016) Association of pretreatment body mass index and survival in human papillomavirus positive oropharyngeal squamous cell carcinoma. Oral Oncol 60:55–60. 10.1016/j.oraloncology.2016.07.003 27531873 10.1016/j.oraloncology.2016.07.003 PMC4991628

[16] Orosz E et al (2017) Comparative miRNA expression profile analysis of squamous cell carcinoma and peritumoral mucosa from the meso- and hypopharynx. Cancer Genomics Proteomics 14(4):285–292. 10.21873/cgp.20039 28647702 10.21873/cgp.20039 PMC5572306

[17] Keller U (2019) Nutritional laboratory markers in malnutrition. J Clin Med 8(6):775. 10.3390/jcm8060775 31159248 10.3390/jcm8060775 PMC6616535

[18] Zhang Z et al (2017) Evaluation of blood biomarkers associated with risk of malnutrition in older adults: a systematic review and meta-analysis. Nutrients 9(8):829. 10.3390/nu9080829 28771192 10.3390/nu9080829 PMC5579622

[19] Bao X et al (2020) Nutritional assessment and prognosis of oral cancer patients: a large-scale prospective study. BMC Cancer 20(1):146. 10.1186/s12885-020-6604-2 32087695 10.1186/s12885-020-6604-2 PMC7036168

[20] Saroul N et al (2018) Which assessment method of malnutrition in head and neck cancer? Otolaryngology–Head Neck Surg 158(6):1065–1071. 10.1177/0194599818755995 10.1177/0194599818755995 29436287

[21] Gunst J, Kashani KB, Hermans G (2019) The urea-creatinine ratio as a novel biomarker of critical illness-associated catabolism. Intensive Care Med 45(12):1813–1815. 10.1007/s00134-019-05810-y 31620835 10.1007/s00134-019-05810-y

[22] Bouillanne O et al (2005) Geriatric nutritional risk index: a new index for evaluating at-risk elderly medical patients2. Am J Clin Nutr 82(4):777–783. 10.1093/ajcn/82.4.777 16210706 10.1093/ajcn/82.4.777

[23] Yanni A et al (2019) Malnutrition in head and neck cancer patients: Impacts and indications of a prophylactic percutaneous endoscopic gastrostomy. Eur Ann Otorhinolaryngol Head Neck Dis 136(3, Supplement):S27-S33. 10.1016/j.anorl.2019.01.001 10.1016/j.anorl.2019.01.001 30846293

[24] Van Schueren Bokhorst-de (1997) Assessment of malnutrition parameters in head and neck cancer and their relation to postoperative complications. Head Neck 19(5):419–425. 10.1002/(sici)1097-0347(199708)19:5%3C419::aid-hed9%3E3.0.co;2-2 9243270 10.1002/(sici)1097-0347(199708)19:5<419::aid-hed9>3.0.co;2-2

[25] Steer B et al (2020) Malnutrition prevalence according to the GLIM criteria in head and neck cancer patients undergoing cancer treatment. Nutrients 12(11). 10.3390/nu12113493 10.3390/nu12113493 PMC7697929 33203000

[26] Cederholm T et al (2019) GLIM criteria for the diagnosis of malnutrition - a consensus report from the global clinical nutrition community. Clin Nutr 38(1):1–9. 10.1016/j.clnu.2018.08.002 30181091 10.1016/j.clnu.2018.08.002

[27] Raber-Durlacher JE et al (2012) Swallowing dysfunction in cancer patients. Support Care Cancer 20(3):433–443. 10.1007/s00520-011-1342-2 22205548 10.1007/s00520-011-1342-2 PMC3271214

[28] Manikantan K et al (2009) Dysphagia in head and neck cancer. Cancer Treat Rev 35(8):724–732. 10.1016/j.ctrv.2009.08.008 19751966 10.1016/j.ctrv.2009.08.008

[29] Logemann JA et al (2006) Site of disease and treatment protocol as correlates of swallowing function in patients with head and neck cancer treated with chemoradiation. Head Neck 28(1):64–73. 10.1002/hed.20299 16302193 10.1002/hed.20299 PMC1380204

[30] Eisbruch A et al (2004) Dysphagia and aspiration after chemoradiotherapy for head-and-neck cancer: which anatomic structures are affected and can they be spared by IMRT? Int J Radiation Oncology*Biology*Physics 60(5):1425–1439. 10.1016/j.ijrobp.2004.05.050 10.1016/j.ijrobp.2004.05.050 15590174

[31] Muscaritoli M, Corsaro E, Molfino A (2021) Awareness of cancer-related malnutrition and its management: analysis of the results from a survey conducted among medical oncologists. Front Oncol 11. 10.3389/fonc.2021.682999 10.3389/fonc.2021.682999 PMC8155516 34055649

[32] Jones L et al (2015) Relationship between alcohol-attributable disease and socioeconomic status, and the role of alcohol consumption in this relationship: a systematic review and meta-analysis. BMC Public Health 15(1):400. 10.1186/s12889-015-1720-7 25928558 10.1186/s12889-015-1720-7 PMC4409704

[33] Brewczyński A et al (2022) Analysis of selected nutritional parameters in patients with HPV-related and non-HPV-related oropharyngeal cancer before and after radiotherapy alone or combined with chemotherapy. Cancers 14(9):2335. 10.3390/cancers14092335 35565464 10.3390/cancers14092335 PMC9101210

[34] Tamaki A et al (2019) Clinical significance of sarcopenia among patients with advanced oropharyngeal cancer. Otolaryngology–Head Neck Surg 160(3):480–487. 10.1177/0194599818793857 10.1177/0194599818793857 30105922

[35] Tan X et al (2015) Obesity and head and neck cancer risk and survival by human papillomavirus serology. Cancer Causes Control 26(1):111–119. 10.1007/s10552-014-0490-3 25398682 10.1007/s10552-014-0490-3 PMC4302732

[36] Tomasoni M et al (2023) The prognostic-nutritional index in HPV-negative head and neck squamous cell carcinoma treated with upfront surgery: a multi-institutional series. Acta Otorhinolaryngol Ital 43(3):170–182. 10.14639/0392-100x-n2358 37204841 10.14639/0392-100X-N2358 PMC10198367

[37] Bruixola G et al (2018) Prognostic nutritional index as an independent prognostic factor in locoregionally advanced squamous cell head and neck cancer. ESMO Open 3(6):e000425. 10.1136/esmoopen-2018-000425 30426973 10.1136/esmoopen-2018-000425 PMC6212680

[38] Tsai MH et al (2020) Clinical significance of pretreatment prognostic nutritional index and lymphocyte-to-monocyte ratio in patients with advanced p16-negative oropharyngeal cancer-a retrospective study. PeerJ 8:e10465. 10.7717/peerj.10465 33344090 10.7717/peerj.10465 PMC7718802

[39] Kono T et al (2017) Pre-therapeutic nutritional assessment for predicting severe adverse events in patients with head and neck cancer treated by radiotherapy. Clin Nutr 36(6):1681–1685. 10.1016/j.clnu.2016.10.021 27847115 10.1016/j.clnu.2016.10.021

[40] Yan L et al (2021) Long-term and short-term prognostic value of the prognostic nutritional index in cancer: a narrative review. Ann Transl Med 9(21):1630. 10.21037/atm-21-4528 34926674 10.21037/atm-21-4528 PMC8640913

[41] Luan C-W et al (2021) Pretreatment prognostic nutritional index as a prognostic marker in head and neck cancer: a systematic review and meta-analysis. Sci Rep 11(1):17117. 10.1038/s41598-021-96598-9 34429476 10.1038/s41598-021-96598-9 PMC8385102

[42] Giles KH et al (2016) Recommended European society of parenteral and enteral nutrition protein and energy intakes and weight loss in patients with head and neck cancer. Head Neck 38(8):1248–1257. 10.1002/hed.24427 27028732 10.1002/hed.24427

[43] Xu Q-Q et al (2022) Effect of prophylactic gastrostomy on nutritional and clinical outcomes in patients with head and neck cancer. Eur J Clin Nutr 76(11):1536–1541. 10.1038/s41430-022-01154-x 35534701 10.1038/s41430-022-01154-x

[44] Espeli V et al (2018) Prolonged versus short-duration use of nasogastric tubes in patients with head and neck cancer during radiotherapy alone or combined chemoradiotherapy. Nutr Cancer 70(7):1069–1074. 10.1080/01635581.2018.1497670 30273007 10.1080/01635581.2018.1497670

